# Lyme Disease Patient Outcomes and Experiences; A Retrospective Cohort Study

**DOI:** 10.3390/healthcare8030322

**Published:** 2020-09-04

**Authors:** Ally G. Rogerson, Vett K. Lloyd

**Affiliations:** Department of Biology, Mount Allison University, Sackville, NB E4L 1G7, Canada; arogerson@mta.ca

**Keywords:** Lyme disease, treatment, patient outcome, retrospective study

## Abstract

Lyme disease is a vector-borne illness caused by *Borrelia* spp. bacterium spread by ticks to humans and other mammals. Despite being prevalent in many regions of the world, there remains considerable uncertainty surrounding many aspects of the disease, and consensus on the most appropriate and effective means of treating the illness remains to be achieved. Recommendations published by the Infectious Diseases Society of America (IDSA) and the International Lyme and Associated Diseases Society (ILADS), the primary guidelines followed by health care professionals treating Lyme disease, diverge in many of their key recommendations, including treatment duration. Given this lack of consensus, surprisingly little research has been conducted on patient outcomes following different treatment approaches. In this study, patient outcomes were evaluated from a cohort of 210 Canadian Lyme disease patients seeking treatment at one US Lyme disease clinic following a treatment regimen conforming to the ILADS treatment guidelines. It was found that the majority of Lyme disease patients at the clinic responded positively to treatment and a significant (*p* < 0.05) decrease in symptoms was observed over time. This study, along with related studies, may help to guide physicians to provide their patients with the most effective care.

## 1. Introduction

Lyme disease is a tick-vectored zoonotic infection caused by pathogenic members of the *Borrelia* genus of spirocheate bacteria [1]. One species, *Borrelia burgdorferi sensu stricto* (*B. burgdorferi ss*), causes the majority of Lyme disease cases in North America, however other members of the genus are also known to cause Lyme disease [1]. *Ixodes scapularis* is the primary tick vector for *B. burgdorferi* in central and eastern North America, whereas in the pacific regions of North America, *Ixodes pacificus* is the primary vector [2]. Ticks acquire *B. burgdorferi* by feeding on infected reservoir species and then transmit the bacteria during subsequent feedings [3]. In addition to infection through tick bites, some evidence exists for transmission through the placenta, by sexual intercourse, and by exposure to the bacteria in blood, urine, or feces from infected organisms [4,5,6], however, further research needs to be done to confirm and quantify non-tick-vectored modes of infection. Climate change, shifting land use patterns, and changes in distribution of reservoir hosts has led to an increase of tick populations, and hence Lyme disease, in some areas of Canada [7]. The years 2009 to 2015 saw a sixfold increase in reported Lyme disease cases [7]. New Brunswick and Nova Scotia, two Maritime Canadian provinces, are seeing a marked increase in Lyme disease cases [8], and the total number of Lyme disease cases in Canada is likely substantially greater than the numbers reported [9]. Diagnosis of Lyme disease remains challenging; the diagnosis is a clinical one supported by tick exposure history and laboratory testing. Vagaries with detection of tick bites and laboratory testing leaves the diagnosis, as well as treatment approaches, fraught with conflicting opinions and evidence. 

If treated with antibiotics in the early stages of the illness, there is consensus that progression of the infection is often avoided [10]. Once the bacterium has entered into the host, it causes an infection in a localized region of the dermis at the site of the tick bite, known as early acute localized Lyme disease [11]. This stage lasts for several weeks while the bacterium multiples in situ [12] and may be associated with the appearance of erythema migrans [13], which is one of the initial diagnostic signs for health care providers. However, this sign presents in only 35–60% of Lyme disease patients [10] and is only useful for some populations [14], making early diagnosis difficult. Other signs of Lyme disease include common nonspecific responses to infection such as unremitting fatigue, fever, aches in the muscles, headaches, and nausea [15]. Although antibiotic treatment shortly after infection has proven to be effective in treating Lyme disease, treatment failure has been reported in 10–35% of patients who are unable to obtain early intervention [16]. If not treated early, for an appropriate time course, or if left untreated, serious multisystem manifestations can develop as the bacteria continue to multiply and migrate to new regions of the body through the blood or the lymph. This is the onset of early disseminated Lyme disease [12,17]. During this stage, early Lyme disease symptoms worsen and cardiac and arthritic symptoms arise as the bacteria invade the vascular system and the connective tissues surrounding the joints [11]. Symptoms attributed to *B. burgdorferi* in the cardiac regions of the body include myocarditis, pericarditis, and atrioventricular heart block [11]. When the bacterium enters the tissues surrounding the joints in the body, it can cause migratory joint pains and synovitis [11]. Late disseminated Lyme disease develops if the infection remains untreated in the months to years after initial infection [12]. During this stage, the bacterium spreads to most organs and tissues of the body and can cross the blood–brain barrier to infect the central nervous system. This creates various severe multisystem manifestations, including neurological symptoms such as migraines, dizziness, sleep disturbances, concentration issues, brain fog, and memory loss [11]. 

To establish an infection in a mammalian host, *Borrelia* needs to evade the host immune response. To do this, the spirocheate employs multiple sophisticated mechanisms including active immune suppression, induction of immune tolerance, antigenic variation, sequestering of the spirochete in immune-privileged tissues, and formation of morphologically and physiologically altered forms able to tolerate antimicrobial treatment [18]. When hostile environments are encountered, such as the presence of antimicrobials, biphasic killing is observed in which some of the bacterial population is killed while persisting subpopulations with pleomorphic phenotypes arise in the form of looped or ring-shaped bacteria, blebs, round bodies, spirochete colonies, or biofilm aggregates [18]. Thus, short-term antibiotic therapy risks generating persisting subpopulations of *B. burgdorferi* that have the ability to alternate between inactive and active forms capable of causing a relapse [18]. There is evidence that a persistent *B. burgdorferi* infection can exist in a variety of mammalian hosts, including mice, rats, hamsters, guinea pigs, gerbils, dogs, monkeys, and baboons [19,20]. Clinical studies also provide evidence of persistent *B. Burgdorferi* infection in humans [21,22,23,24]. Using various detection methods including microscopy, histopathological and molecular testing, one study found evidence of ongoing spirochetal infection despite antibiotic treatment, cultured from body fluids and tissues from a group of patients with persistent Lyme disease symptoms [25]. Another study found similar results using immunoelectron microscopy and PCR to detect *Borrelia* in plasmas and cerebrospinal fluid from a group of Lyme disease patients experiencing persistent symptoms 4 to 68 months after antibiotic treatment [26]. Additionally, uninfected ticks can become infected after placement on a post-treatment symptomatic individual, further suggesting the presence of a persistent infection [27]. Evidence against persistence tends to be indirect and based on studies showing no benefit to antibiotic retreatment of symptomatic post-treatment individuals. Reports of failure to identify *Borrelia* using molecular testing are limited by difficulty in publishing negative results, however, Li et al. [28] did report that synovial fluid and tissue from post-treatment individuals found persisting DNA but not RNA. This was interpreted as evidence that persisting *Borrelia* were dead [28], although, a metabolically quiescent state cannot be excluded as an explanation of these results. 

Two primary treatment guidelines exist for the management of Lyme disease: guidelines published by the Infectious Diseases Society of America (IDSA) and guidelines published by the International Lyme and Associated Diseases Society (ILADS). The majority of health care providers in Canada follow a conservative version of the IDSA guidelines endorsed by the Association of Medical Microbiology and Infectious Diseases of Canada. A number of recommendations differ between the ILADS and the IDSA published guidelines and, therefore, a consensus has yet to be agreed upon by the medical community [10,29]. Among these differences are the proposed causes of persistent symptoms. While IDSA guidelines attribute persisting symptoms to tissue damage or an autoimmune response [30,31], ILADS guidelines recognize evidence for persistent *B. burgdorferi* infection. As ongoing symptoms might arise from unresolved infection, these guidelines support an open-ended treatment approach for bacterial elimination [10,19]. While animal model and research evidence for *Borrelia* persistence is fairly abundant, clinical evidence for the efficacy of longer-duration treatment is based on a limited number of studies [10]. The paucity of clinical studies supporting either set of guidelines is further exacerbated by inconsistencies in enrolment criteria, interventions, outcome measures, and conclusions [10]. As such, extensive and rigorous additional research and clinical trials on Lyme disease treatment approaches are needed [10].

It has been found that a large number of Canadian Lyme disease patients are dissatisfied with their Lyme disease treatment in Canada and therefore seek treatment options internationally, often in the United States where they are able to receive treatment following ILADS treatment guidelines [32]. Despite Lyme disease being the most common tick-vectored disease in the western hemisphere, and despite the projected future increase in cases, studies of patient outcomes following treatment remain sparse, illustrating the critical need for evidence-based research to guide physician treatment regimens in Canada. The aim of this research was to evaluate the efficacy of a treatment approach involving long-term use of multiple antimicrobials from one American Lyme disease clinic treating patients following ILADS treatment guidelines. Data from symptom charts of 210 Canadian Lyme disease patients treated at this clinic were analyzed for changes in the number and nature of symptoms over treatment time, and patients were invited to provide additional information on their illness and treatment through a survey. We find that the majority of patients responded positively to treatment and a significant (*p* < 0.05) decrease in symptoms was observed over time. 

## 2. Materials and Methods 

### 2.1. Participants and Setting 

**Figure 1:**
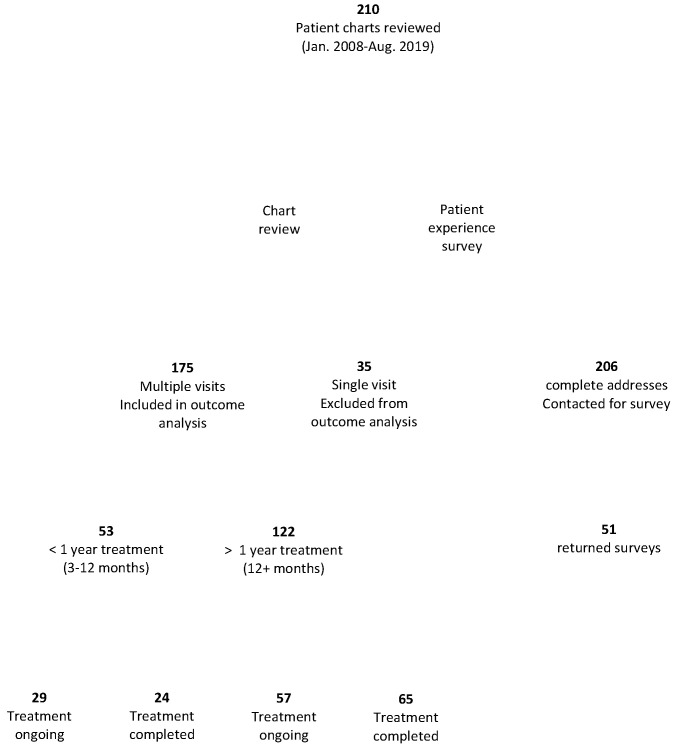
Study design. The study design and the number of patients participating in the survey and included in the chart review is shown.

All subjects in this study were residents of Canada who received or were receiving treatment by Dr. Richard Dubocq at a Family Medicine and Geriatrics clinic in Albion Maine, a physician with special interest in Lyme disease. Charts were reviewed from patients seen between January 2008 and August 2019, with no known exclusions. All data were collected by the physician’s assistants in August 2019. Information collected included patients’ age, gender, symptoms, and health history. No Canadian medical files were reviewed. Although some files contained letters of referral containing some patient history, these and any Canadian medical documents were all patient-provided. These documents were not present in all files and were heterogeneous in completeness and format. In addition to chart review, a survey seeking additional information on tick exposure, diagnoses, and patient experiences at both the U.S. Lyme disease clinic and with Canadian health care providers was designed by the research team and sent to patients by clinic staff. The survey was sent by the clinic to 206 of the 210 patients with current addresses on file, and the completed surveys were returned directly to the researchers. Of the 206 contacted patients, 51 (25%) returned completed surveys (Figure 1).

Patients included in this study were self-referred; although in some cases treatment under ILADS guidelines had been recommended by their Canadian health care practitioners, no patients were formally referred. All patients included in this study were given a clinical diagnosis of chronic/disseminated Lyme disease with a long-standing duration of symptoms and/or likely tick exposure and/or supportive laboratory diagnostics by the treating physician. This cohort would not be expected to be representative of all Canadians with Lyme disease; the cost and difficulty of seeking treatment outside of Canada would select for patients most motivated to pursue treatment and with the means to do so [32]. Presumably those diagnosed and treated appropriately for acute Lyme disease in Canada would be under-represented in this study, hence the patient cohort represents primarily those with complex cases of chronic/disseminated Lyme disease. Some patients had alternate, as well as additional, diagnoses by health care providers in Canada. Some had had treatment for Lyme disease, varying from a single dose of doxycycline to several months of oral antibiotics, others had not previously been treated for Lyme disease, however, full details of the type and timing of early treatment were not available for all patients so is not considered in this study. Permission for secondary use of anonymous data and for collection of survey data was approved by the Mount Allison University Research Ethics Board (Research Ethics Protocol #102622). 

### 2.2. Study Design

Symptom charts (Table 1) consisting of specific symptoms in various categories were completed by each patient at each consultation, which occurred at approximately 3-month intervals. A total of 44 multisystem symptoms of Lyme disease were recorded (Table 1).

Patients included in this analysis were those who had at least two consultations at the clinic between January 2008 and August 2019 (Figure 1). The multiple-consult subset of patients was then further divided into groups based on treatment time, measured by the number of consultations the patients had had at the clinic (Figure 1). These groups were then subdivided into those that had presumably completed treatment and those with ongoing treatment. Those considered to have completed treatment had no consultations at the clinic for at least one year prior to data collection, and those considered to be in ongoing treatment had visits to the clinic within the past year.

(a) *Patients completed treatment in less than a year.* This subset of patients had either two or three consultations at the clinic before ceasing visits. This group had no consultations at the clinic for at least one year prior to data collection.
(b) *Patients with ongoing treatment <1 year.* This subset of patients had either two or three consultations at the clinic with their most recent consultation within one year prior to data collection.
(c) *Patients completed treatment >1 year.* These patients were those who had had treatment regimens lasting more than 1 year but had no consultations at the clinic for at least one year prior to data collection.
(d) *Patients with ongoing treatment >1 year.* This subset of patients was at the clinic for more than one year with their most recent consultation at the clinic within one year prior to data collection.

Patient response to treatment was measured by the change in the number of symptoms, from the initial visit to the final or most recent visit for each of the four patient groups. Change in total number of symptoms and changes in number of symptoms within specific categories were assessed. Change in number of symptoms at first consultation compared with most recent consultation to the clinic was calculated using the following equation:

Change in symptoms over time = Number of symptoms at most recent visit—Number of symptoms at initial visit.

High responders to treatment were defined as having the greatest change and were represented by data points less than −10 (Δ > 10 symptoms), moderate responders were represented by data points ranging from −9 to 0 (Δ 0–9 symptoms), non-responders were represented by data points at zero (Δ 0 symptoms), and patients who were worse after treatment were represented by positive data points (Δ n+ symptoms).

In order to determine if each body system was differentially affected by treatment, change in number of patients that had each symptom, in each symptom class, at first visit compared with most recent visit to the clinic was calculated for all patients in the study. The following equation was to calculate change in number of patients with each symptom: 

Change in number of patients with each symptom = Number of patients with symptom at their most recent visit—Number of patients with symptom at their initial visit 

### 2.3. Statistical Analysis 

In order to determine if symptom number was changing over time (measured by the number of consultations) for each of the four patient groups, data was analyzed using a generalized linear mixed model (alpha value = 0.05) in R software (Version 3.6.2, “Dark and Stormy Night”). A generalized linear mixed model work well with unbalanced repeated measures and longitudinal data as it assumes a continuous outcome variable that is related to a set of explanatory variables following a linear trend [33]. This method also allows for lack of independence between observations, as is true for repeated measures studies, making it the optimal approach to analyze trends in multiple observations on a single subject with flexibility [33]. The Poisson distribution is useful to model count data such as the measures used in this study, because it specifies probability for only integer values [34]. 

## 3. Results

### 3.1. Patient Demographics

At the time of data collection via chart review, there were 210 Canadian patients who were currently receiving or had previously received treatment from the U.S. Lyme disease clinic between January 2008 and August 2019, with the majority of patients seen after 2013. In addition to chart review, to address the patient experience, we invited patients to complete a survey on their experiences with treatment at the clinic and with other health care providers (Figure 1). 

The majority of the 210 patients included in this study were from Canadian Maritime provinces, with most being from New Brunswick (NB; 61.0%) and Nova Scotia (NS; 32.4%). Other patients included were from Quebec (QC; 6.7%), Prince Edward Island (PE; 1.4%), Ontario (ON; 1.0%), Newfoundland (NL; 0.5%), and Manitoba (MB; 0.5%), Patients’ demographic and clinical data included age, sex, comorbidities, history of health, and tick exposure history (Table 2). Those responding to the survey were reflective of the demographics of the larger cohort whose charts were reviewed. In both the chart review and additional survey, 59% and 41% of the respondents identified as female and male, respectively. Survey respondents reflected the same geographic regions (67% NB, 29% NS, 4% PE) as the chart review. Chart review indicated 49% of patients had known tick bite or rash versus 43% in the survey. The similarity in demographic information between these two measures of the same patient cohort suggests that the survey results represent an unbiased selection of the larger group of patients studied, and additionally provides confirmation of chart completeness. Although representative, the survey return rate was 25%. This is consistent with other surveys of patients with significant chronic illness [35] but makes full analysis of the survey results more suitable for qualitative analysis, which will be reported elsewhere. 

This study focused on patient outcome, in part as a function of treatment time. Patients received care at the clinic for varying lengths of time, although all treatment durations were longer than the standard IDSA treatment duration. Of the 210 Canadian patients, 35 (15 males and 20 females; 17%) of the patients visited the clinic only once (Figure 1, Table 3). This group was excluded from this analysis as this group includes those not given a diagnosis of Lyme disease. An unknown proportion of this group would also include those who chose not to pursue treatment at the clinic for, presumably, diverse but undocumented reasons. The patient experience survey allowed us to explore reasons for terminating treatment in more detail, albeit with the caveat that only a proportion of patients completed the survey and only a minority of patients did not pursue treatment. Both patients who did and patients who did not pursue treatment were invited to complete this survey. Of the 51 survey respondents, the majority, 40, were still continuing treatment. Of the 11 who had terminated treatment, reasons noted were medication side effects (6), treatment completion (5), financial barriers (4), a preference for treatment by another (U.S.) physician (1), and pressure to discontinue treatment by Canadian health care professionals (1). Multiple reasons were provided by some respondents. 

To focus on patient outcomes, the number and types of symptoms reported by patients with two or more consultations were collated over treatment time. These patients were divided into four groups based on treatment time and completion; those treated for 3–12 months (<1-year treatment treatment) and those treated for 12+ months (>1-year treatment). Each of these groups were then subdivided into those that had presumably completed treatment based on either clinic notes or no return consultations for at least one year prior to data collection and those with ongoing treatment. 

At the time of data collection, some patients had completed treatment (42.4%), while some were still undergoing treatment (41%) and a small percentage had not pursued treatment past the first consultation (17%) (Table 3). There was a greater number of females (62%) than males (38%) receiving treatment at the clinic (Table 3).

### 3.2. Symptoms Reported

**Figure 2:**
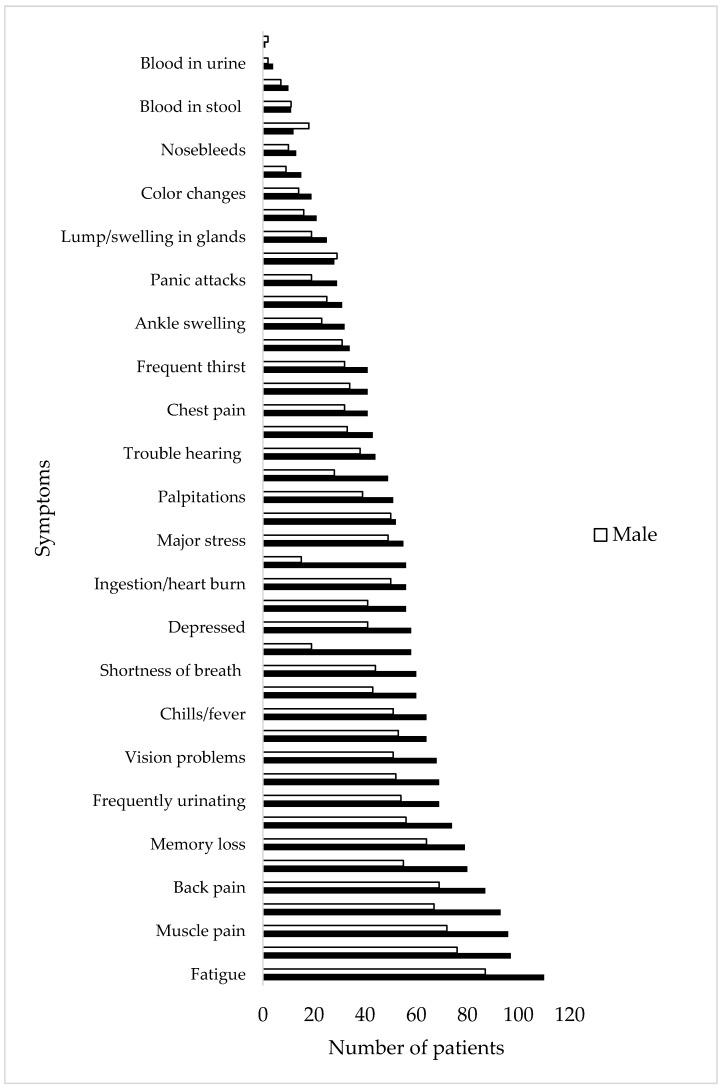
Number of males and females with each symptom at their initial visit to the US Lyme disease clinic (N = 210).

The number of patients who had each symptom in each body system was totaled to determine the most commonly observed symptoms upon presentation to the clinic. Back pain, dizziness, muscle pain, joint pain, and fatigue were the five most commonly observed symptoms (Figure 2). The average number of symptoms for females and males at the start of treatment was 19 and 17, respectively.

### 3.3. Determination of Patient Response to Treatment 

In order to analyze patients’ response to treatment, the change in the number of symptoms between the patients’ initial visit to the clinic and their last or most recent visit was calculated for each of the four patient groups; the initial number of symptoms was subtracted from the final number of symptoms so that negative values reflect fewer symptoms, positive values represent increased numbers of symptoms, and 0 represents no change in total symptom number. 

**Figure 3:**
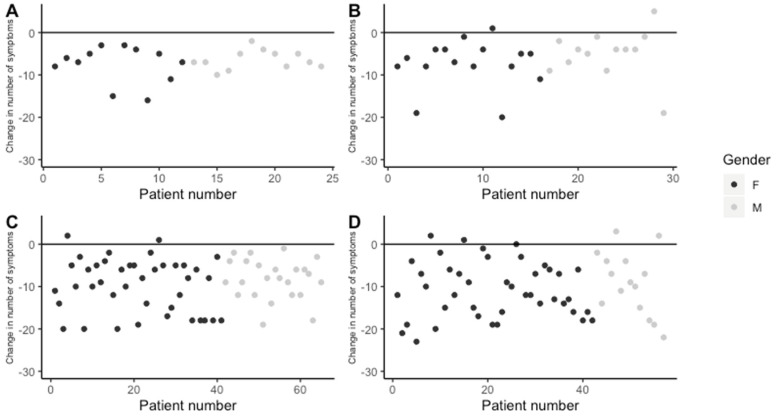
Change in number of symptoms reported between initial and final/most recent visit. (**A**) Patients with completed <1 year treatment, (**B**) patients with ongoing <1 year treatment, (**C**) patients with completed >1 year treatment, and (**D**) patients with >1 year ongoing treatment.

All patients in the <1 year completed treatment group responded to treatment, reporting fewer symptoms at the end of treatment than at the beginning (Figure 3A). In the group of patients in ongoing <1 year treatment, the majority of patients were responding positively to treatment at the time of data collection, although two patients (one male and one female) (6.9%) responded negatively (Figure 3B). Among patients who completed >1 year treatment at the clinic, the majority of patients responded positively, while two patients (females) (6.1%) responded negatively to treatment (Figure 3C). In the ongoing >1 year treatment group, the majority of patients were responding positively at the time of data collection, although four patients (two males and two females) (7%) had responded negatively and there was one patient (female) (1.7%) who had the same number of symptoms as upon initial presentation, although the symptoms were different (data not shown; Figure 3D). 

**Figure 4:**
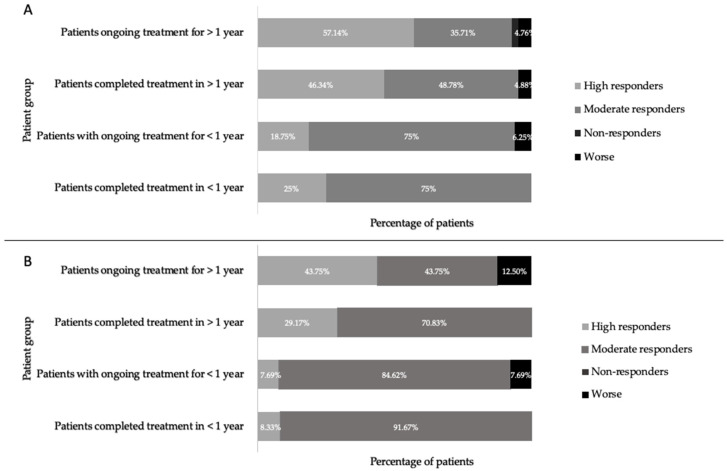
Percentage of females (**A**) and males (**B**) who were high responders (Δ > 10 symptoms), moderate responders (Δ 1–9 symptoms), non-responders (Δ 0 symptoms), and worse (Δ n+ symptoms) after the start of treatment, in patients who ceased treatment early, patients ongoing <1 year of treatment, patients ongoing >1 year of treatment, and patients who completed treatment at the US Lyme disease clinic.

**Figure 5:**
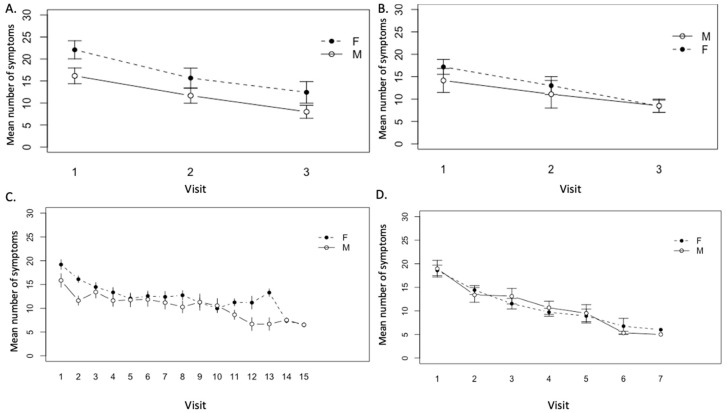
Mean number of symptoms over visits to the Lyme disease clinic in (**A**) patients who completed <1 year treatment (with three visits) (*n* = 12 females, 12 males), (**B**) patients with ongoing <1 year treatment (*n* = 16 females, 13 males), (**C**) patients who completed >1 year treatment (*n* = 41 females, 24 males), and (**D**) patients with ongoing >1 year treatment (*n* = 42 females, 15 males).

The extent to which individual patients responded to treatment was assessed by the scale of symptom resolution. Those with a change in more than 10 symptoms (Δ > 10 symptoms) are termed high responders, those with a change in 1–9 symptoms (Δ 1–9 symptoms) are termed moderate responders, and those with no change (Δ = 0) are termed non-responders. Comparing the response of patients to treatment in each group, it was found that the majority of patients with high responses, both male and female, were those in the completed long-term and the ongoing long-term groups (Figure 4). This is not simply an artefact of higher symptom numbers in a group or gender; all groups had between 14 and 23 symptoms upon initial presentation (Figure 5). When comparing the response of patients to treatment between genders it was found that a greater percentage of females were high responders, and although the average symptom number was higher in females than in males, this difference was not statistically significant (Figure 5).

### 3.4. Determination of Average Change in Symptom Number over Time

To assess variation in number of symptoms between visits to the clinic over time, a generalized linear mixed model analysis was used. In all patient groups, a downward trend in mean number of symptoms was observed in both males and females. 

In the patient groups that completed <1 year treatment, the difference in the number of symptoms between three visits was significant (*p* < 0.01) (Figure 5A). In this patient group, the average female and male symptom number at the start of treatment was 22.0 and 16.6, respectively, and the number of symptoms reported at each clinic visit was higher for females than for males, however, this difference was not significant (*p* = 0.28) (Figure 5A). A continuous downward trend in number of symptoms was similarly observed in both males and females in the patient group with ongoing <1 year treatment (Figure 5B). At the start of treatment, females and males reported an average of 17.2 and 14.6 symptoms, respectively. Over the course of treatment, the decrease in mean number of symptoms between visits was significant (*p* < 0.001), but there was no significant difference between males and females in symptom number and response (*p* = 0.23) (Figure 5B). Similarly, a continuous downward trend in mean number of symptoms was observed in both males and females over visits to the clinic in patients who completed >1 year treatment. The difference in mean number of symptoms at each visit over the course of treatment was determined to be significant (*p* < 0.001) (Figure 5C). At the start of treatment, females and males in this group reported an average of 19.2 and 16.6 symptoms, respectively. Again, females in this group had a greater number of symptoms in the majority of visits compared with males, however, differences between genders were not significant (*p* = 0.11) (Figure 5C). A similar pattern was seen in patients in the ongoing >1 year treatment group; a continuous and statistically significant downward trend in the number of symptoms was observed in both males and females over visits to the clinic (*p* < 0.001) (Figure 5D). Females in this patient group had a greater number of symptoms in the majority of visits compared with males, although the average incoming symptom number was 17.8 and 18.5 for females and males, respectively. Again, these differences were not significant (*p* = 0.11) (Figure 5D).

### 3.5. Effect of Treatment on Specific Symptoms 

**Figure 6:**
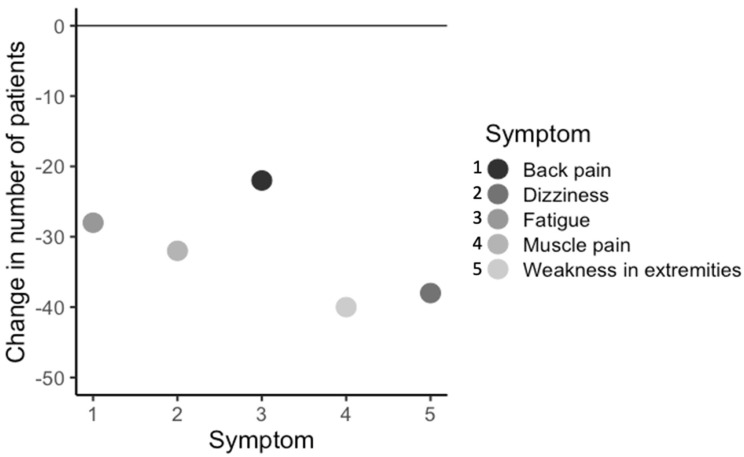
Change in number of patients (*n* = 175), within all patient groups, with the most commonly observed symptoms, before and after completion or most recent treatment.

As the total number of symptoms decreased, it was of interest to see if some of the 44 symptoms in each of the 13 body systems measured were differentially affected by treatment. To assess this, the change in number of patients (N = 175) who had each symptom before and after the start or completion of treatment was calculated, and symptoms were categorized as highly responsive to treatment (>20 patients responding by decreasing symptoms), moderately responsive (10–20 patients responding), slightly responsive (1–9 patients responding), non-responsive (0 patients responding), or negatively responsive (an increase in patients with that symptom after treatment). Out of 44 symptoms, 26 symptoms responded highly to treatment, 9 symptoms responded moderately to treatment, 8 symptoms slightly responded to treatment, and no symptoms failed to respond to treatment, although 1 symptom (lumps and swelling in the glands) got worse after the start or completion of treatment. Importantly, the five most commonly observed symptoms were highly responsive (Δ > 20 patients) to treatment at the clinic (Figure 6).

**Figure 7:**
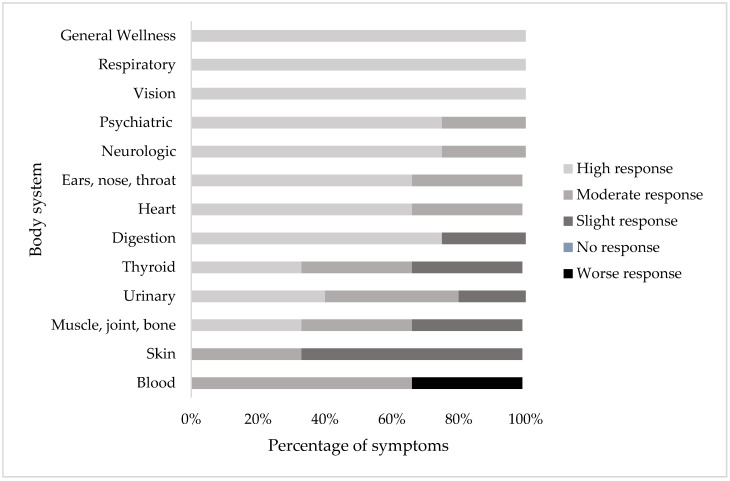
Percentage of symptoms in each body system that responded highly (Δ > 20 patients), moderately (Δ 10–20 patients), slightly (Δ 1–9 patients), no response (Δ 0 patients), or a worse (Δ n+ patients) response in patients before and after the start or completion of treatment in patients with two or more consultations (N = 175).

Each of the 44 symptoms was categorized into 13 body system groups (Figure 7). For the general wellness, respiratory, and vision categories, all symptoms were observed to be highly responsive to treatment at the clinic (Δ > 20 patients showing improvement). Symptoms in the heart, psychiatric, neurological, and ENT categories were observed to be highly or moderately responsive to treatment. The majority of symptoms in the digestion category were highly responsive to treatment, while one symptom (blood in stool) was slightly responsive to treatment. In the symptom categories urinary, thyroid, and muscle and joint and bone, the majority of symptoms were observed to be highly or moderately responsive to treatment, while some symptoms were observed to be only slightly responsive to treatment. In the skin symptom category, one symptom was highly responsive to treatment (lesions) while the others were slightly responsive. In the blood symptom category, some symptoms were moderately or slightly responsive to treatment while one symptom (lumps and swelling in the glands) was observed to get worse. Thus, while most symptoms in most body system categories improved, some appeared to be more responsive to treatment in that they showed improvement in more patients than others. 

## 4. Discussion

This study focused on determining the responses of Lyme disease patients with a clinical diagnosis of chronic Lyme disease to a treatment regimen based on ILADS treatment guidelines. Patient outcomes for 210 Canadian patients seeking treatment between January 2008 and August 2019 at a single medical clinic in the United States that offers treatment following the ILADS treatment guidelines were evaluated. As an alternative to treatment regimens offered in Canada, which are generally based on the IDSA treatment guidelines, some Canadian patients seek treatment regimens based on ILADS treatment guidelines, which are more readily available in the United States [32]. Patients included in this study either had not been treated for tick-vectored diseases or antibiotic treatment of short duration had taken place, but wellness had not been restored to the patients’ satisfaction. 

### 4.1. Patients’ Response to Treatment 

After calculating the change in number of symptoms patients had at their initial and most recent visit to the clinic, the majority of patients were found to respond positively to treatment. There was a significant decrease in number of symptoms over time and visits to the clinic in each of the patient groups, those who had completed treatment and those in ongoing treatment, both less than and greater than 1 year. Patients reported an average of 17 and 19 symptoms for males and females, respectively, at the start of treatment. This fell to an average of 7 and 9 for males and females, respectively, for all patient groups, by the end of treatment. A decrease in 10 symptoms reflects a significant reduction in self-assessed illness. Nevertheless, no patients reported zero symptoms at completion of treatment. The residual symptoms generally, but not always, included the original symptoms, however the percentage of those with original symptoms generally decreased with treatment duration. For those in the ongoing <1 year, completed <1 year, ongoing >1 year, and completed >1 year groups, the percent eliminating all original symptoms was 14%, 8%, 11%, and 17%, respectively. Similarly, the percent of original to total symptoms decreased in the same groups from 51%, 42%, 45%, and 37%, respectively. However, in this study, five females and three males (4.3% of patients) reported more symptoms after the start of treatment. This might be attributed to variability in the progression of the illness, treatment side effects, pre-existing comorbidities, or new conditions. 

In patients treated for less than 1 year, whether or not treatment was ongoing, the majority of patients were moderately responsive to treatment with a decrease in 1–9 symptoms, while a smaller percentage of patients in this group responded markedly to treatment, reporting a change in more than 10 symptoms. When comparing these results to the patient group that had undergone treatment for greater than 1 year, both ongoing and completed treatment groups, a greater percentage of patients in the groups treated over a longer duration showed marked improvement, defined as a change in greater than 10 symptoms. It is difficult to make general comparisons between the groups as each patient would have had a different number and type of symptoms at their initial visit to the clinic, which may have an effect on the number of symptoms that could change over time and with treatment. However, with these caveats, the results presented here indicate that longer treatment durations, over 1 year, with the protocol used at this clinic may have better symptom outcomes compared with treatment durations of less than 1 year. When comparing the results of this study to previous studies that used similar treatment regimens, comparable results were observed. A study conducted in 1997 on 277 Lyme disease patients using long-term tetracycline therapy found a larger percentage of patients whose conditions improved after longer durations of the treatment compared with their state earlier on in the treatment [36]. Similarly, a study comparing results from a group of 30 Lyme disease patients treated with antibiotics for 14 days to a group of 30 Lyme disease patients treated with antibiotics for 100 days found that a greater percentage of patients showed good or excellent treatment response with a lower rate of clinical relapse after treatment for 100 days compared with treatment for 14 days [37].

A number of studies have compared short- and longer-duration treatment regimens. Some of these studies have found that the benefits realized from extended or additional treatment did not differ statistically from placebo treatment [38,39,40], while others have found improvement in some symptoms, but not in others [19,41,42]. Resolving these discrepant findings is likely to be complex, and their relevance to the current study is unclear as the disease duration before diagnosis, treatment modalities upon diagnosis, and treatment type and duration in these trials are both varied and not routinely available to Canadian patients.

### 4.2. Gender-Based Differences in Presentation and Recovery

In this study, there were more females than males seeking treatment and, on average, females in these two groups had a greater mean number of symptoms at each visit to the clinic, although, the difference in symptom number between genders in each treatment group was not significant. There is extensive literature showing that females are more likely to seek health care than males. However, the Public Health Agency of Canada and other public health agencies report a greater percentage of males with Lyme disease than females [7,8], so one might have expected a more equal number of males and females seeking treatment. It has been reported that males are more likely to present with positive Lyme disease test results compared with females [43], which might lead to earlier and more successful treatment. Jarefors et al. [44] found that females with Lyme disease had significantly higher spontaneous secretion of cytokines compared with males, which is indicative of a greater inflammatory response and potentially inflammation-related symptoms. An intensified inflammatory response to *B. burgdorferi* may also influence the female response to treatment; females were found to be less likely to respond positively to long-term treatment compared with males [36]. Notwithstanding this difference, males and females were approximately equally represented in the group of patients who ceased treatment early and in the group of patients with ongoing treatment <1 year at the clinic. Further, trends in number of symptoms over visits at the clinic were similar in in both males and females. The gender-based differences in the patient population presenting with chronic Lyme disease is deserving of wider and further study. 

### 4.3. Response to Treatment at the Body Systems Level

The most commonly observed symptoms of the patients in this study included back pain, dizziness, fatigue, muscle pain, and weakness in the extremities. This finding corresponds with those in other studies; fatigue, joint pain, difficulty in focusing, muscle pain, and memory were rated among the most common and/or severe [16,38,40,45,46,47,48]. After calculating the change in number of patients with each symptom before and after treatment, it was found that the majority of symptoms improved in most of the symptom categories. This included the most commonly observed symptoms, with the one exception to the overall general improvement in the “blood” symptom category. 

Symptoms will vary depending on the tissue or organ most heavily impacted, by patients’ genetic and immunological variability, as well as many other factors [49]. Additionally, one symptom class may reflect primarily active infection, while others may reflect an autoimmune response, or damage to the tissue. These primary etiological events will respond differently to treatment and have different recovery trajectories. Indeed, the differential response of various symptoms may contribute to different findings on the efficacy of longer-term treatment for Lyme disease; if a symptom class that is not highly responsive is measured, no apparent improvement will be recorded. Regardless, of the research implications of this observation, from the patient perspective, in all cases it is important to note that these measures are averages and do not represent the experiences of every patient or their state of wellness at all times, merely those at the time they reported symptoms. To extend this study, it would be valuable to obtain the perspective and experiences of patients in more detail, both with their treatment at this Lyme disease clinic and with other health care providers. A longitudinal study following treatment completion would also be valuable to assess the stability and completion of their recovery.

### 4.4. Limitations of the Study

Retrospective cohort studies are necessarily limited in the level of control the researchers have over lifestyle and other variables [50,51]. As a retrospective patient outcome study, there was no untreated control patient group, so it is not formally possible to eliminate the possibility that the symptoms spontaneously resolved over time as opposed to the treatment being responsible for the reduction in symptoms. Although relatively few studies monitor disease progression in known, but untreated, Lyme disease patients, one study, looking at 13 untreated Lyme disease patients with persistent symptoms for greater than 12 weeks, found that patients continued to have symptoms with no relief for a period of 3 months to 5 years after initial onset of symptoms [44]. Another study looking at 55 untreated Lyme disease patients with persistent symptoms for an average of 6 years found that 80% of the patients in this cohort experienced continual symptoms over time [15]. These findings support the widely accepted observation that, if left untreated, Lyme disease will continue to cause multisystem manifestations in patients that will not be resolved without intervention [10]. 

Additionally, in this study, as in most treatment outcome studies and in the general population, the patient population was diverse. Lyme disease affects all ages and genders [16]. Heterogeneity can exist in severity of the disease among different individuals, which may impact both the patients’ relative adherence and responses to treatment. Similarly, patients’ background, prior health care, and comorbidities will affect their health. Further, patients may have different levels of vulnerability to adverse side effects from the treatment. Finally, in Lyme disease, delays in diagnosis would also have a significant impact on disease severity and response to treatment [16]. To, somewhat, counter this heterogeneity, this study was conducted on patients seeking treatment from only one U.S. Lyme disease clinic and patients were treated by only one physician at the clinic. Nevertheless, patient treatment was customized to each patient; with each receiving medications and dosages specific to their diagnosis and medication tolerances, so this is still a source of potential variability in response and a homogeneous response is unlikely under these circumstances. While the patient population was diverse, it would not be expected to be representative of all Canadians with Lyme disease due to the cost and difficulty of obtaining treatment outside of Canada [32]. Johnson et al. [16] report the treatment outcomes of a large number of American patients. The response of the Canadian patients in this study is similar in some respects; approximately the same proportion responded very well to treatment, however, the proportion of patients noting a tick bite and EM was lower in this patient cohort than reported in [16]. This might suggest less community-level awareness of ticks among the Canadian population, which could lead to a higher proportion of patients not arresting disease progression at early stages. Finally, all data used in this study was primarily patient-reported and there is a level of subjectivity that should be considered as an expected source of additional variability; nevertheless, this also makes this data directly relevant to the patient experience. The influence of these factors on treatment outcomes, in particular whether the results of this study can be extended to other Lyme disease patients undergoing similar treatment regimens elsewhere, is an important area requiring further study. 

## 5. Conclusions

Ongoing disputes within the medical community concerning most aspects of Lyme disease have resulted in fewer studies on this illness in comparison with other diseases, infectious and otherwise, despite Lyme disease being an illness affecting many individuals in many regions of the world [16]. The findings presented in this study show that treatment, as practiced by one physician at a single Lyme disease clinic in the USA following ILADS treatment guidelines, was effective in relieving symptoms in a cohort of Canadian patients. More research is needed on patient responses to treatment, both retrospective and prospective cohort studies, following both of the treatment guidelines designed to aid physicians in developing treatment regimens. The lack of this information has resulted in uncertainty for both patients and those treating them [47], a situation that needs to be resolved as the number of infections increases.

## Figures and Tables

**Table 1:** Symptoms recorded by patients at each consultation to the US Lyme disease clinic.

Category	Symptoms
General wellness	weight fluctuations, night sweats, fatigue, Chills
Vision	vision problems, eye pain
Digestion	ingestion/heart burn, nausea/vomiting, vomiting blood, blood in stool
Heart/Circulation	chest pain, palpitations, ankle swelling
Urinary	blood in urine, pain when urinating, weak urine flow, frequent urination, urinary leaking
Thyroid/Endocrine	hot flashes, frequent thirst, hair changes
Skin	lesions, itching/rash, color changes
Lungs/Breathing	shortness of breath, wheezing, frequent cough
Blood/Lymph	bruising easily, protracted bleeding
Neurological	weakness in extremities, dizziness, difficulty with balance, memory loss
Ear, Nose, Throat (ENT)	trouble hearing, sinus trouble, nosebleed
Muscles, Joints, Bones	joint pain, muscle pain, back pain
Psychiatric	panic attacks, anxious, depressed, feeling major stress

**Table 2:** Demographic characteristics of 210 Canadian patients treated at one US Lyme disease clinic.

Patient Characteristic	Number	Percentage (%)
Age, years		
<60	116	55.2
>60	94	44.8
Sex		
Male	86	41.0
Female	124	59.0
**Presence of comorbidity**		
Anemia	40	19.0
High blood pressure	52	24.8
Heart murmur	23	11.0
Asthma	24	11.4
Diabetes	15	7.14
Irritable bowel syndrome	78	37.1
Yellow jaundice	9	4.29
Seizures	13	6.19
Nervous condition	28	13.3
Gout	9	4.29
Arthritis	64	30.4
Migraines	96	45.7
**History of health**		
Heart attack	11	5.23
Stroke	7	3.33
Pneumonia	47	22.4
Ulcer	18	8.57
Hernia	19	9.05
Cancer	27	12.4
Blood clot	9	4.29
Kidney infection	47	22.4
Kidney stones	42	20.0
Fractures	63	30.0
**Tick exposure history**		
Known tick bite	56	26.7
erythema migrans (EM) rash	46	21.9

**Table 3:** Treatment status of Canadian patients at the US Lyme disease clinic from January 2008 to August 2019.

Patient Group	Patients		
	Female Number	Male Number	Total Percentage (%)
Only one consultation (excluded from chart review)	20	15	17%
Patients who completed treatment in <1 year (3–12 months)	12	12	11%
Patients with ongoing treatment for <1 year (3–12 months)	16	13	14%
Patients who completed treatment >1 year (12+ months)	41	24	31%
Patients with ongoing treatment for >1 year (12+ months)	42	15	27%
